# An Advisory and Treatment Centre

**Published:** 1920-10-23

**Authors:** 


### An Advisory and Treatment Centre.

The Women's Imperial Health Association is understood to be organising a scheme by which girl employees, clerks, typists, etc., who are not in a position to pay the higher consultation fees, will be enabled to secure the skilled medical advice at what is to be known as the "Girls' Advisory and Treatment Centre," which it is proposed shortly to open in Hanover Square.

